# Well-being and sense of security of intubated patients in intensive care units: a patient co-constructed dedicated scale

**DOI:** 10.1186/s13054-026-05923-z

**Published:** 2026-03-07

**Authors:** Laetitia Bodet-Contentin, Hélène Lecompte, Adrien Lociciro, Nancy Kentish Barnes, Hélène Messet, Misylias Bouaoud, Justine Cibron, Nicolas Chudeau, François Barbier, Carole Haubertin, Laurent Poiroux, Benedicte Sautenet, Wissam El Hage, Amélie Le Gouge, Julie Leger, Jean-Benoit Hardouin, Stephan Ehrmann

**Affiliations:** 1 Médecine Intensive Réanimation, CHRU Tours, INSERM CIC 1415, CRICS-Triggersep F- CRIN research network, Tours, France; 2 INSERM, SPHERE, UMR1246, Université de Tours et Nantes, Tours et Nantes, France; 3 CHRU Tours, Médecine Intensive Réanimation, Tours, France; 4 Medical Intensive Care, Famiréa Research Group, AP-HP Nord, Saint Louis Hospital, Paris, France; 5 CH Le Mans, Médecine Intensive Réanimation, Tours, France; 6 CHU Orléans, Médecine Intensive Réanimation, Tours, France; 7 CHU Angers, Médecine Intensive Réanimation, Tours, France; 8 Nursing Department, Health Faculty, Univ Angers, Univ Rennes, EHESP, Irset (Institut de recherche en santé, environnement et travail) - UMR_S 1085, SFR ICAT, Inserm, Angers, F- 49000 France; 9 Department of Nephrology, CHU de Tours, Tours, France; 10 F-CRIN INI-CRCT (Cardiovascular and Renal Clinical Trialists), Inserm UMR1246 SPHERE, Université de Nantes, Université de Tours, Tours, France; 11 Imaging Brain & Neuropsychiatry iBraiN U1253, Université de Tours, INSERM, Tours, 37032 France; 12 INSERM CIC1415, CHU de Tours, Tours, France; 13 Centre d’étude des pathologies respiratoires, U1100, INSERM, Université de Tours, Tours, France

**Keywords:** Patient centered outcome, Quality of life, Security, Intubated, Intensive care, Mental health

## Abstract

**Purpose:**

Intensive care unit (ICU) patients endure significant stress due to their critical condition, communication difficulties, and the hostile environment. Despite efforts to humanize ICUs, there is a lack of real-time assessment tools for patient well-being and sense of safety. The aim of this project was to develop a scale assessing the feeling of well-being and safety in real time among intubated patients.

**Methods:**

A systematic review was performed to identify study outcomes evaluating well-being and sense of security. Results were used to organize focus groups and explore patients’ experiences during their ICU stay. The scale was then developed via Delphi methodology by a patient-professional group. The scale was validated through patient interviews for face validity and implementation in a multicentric French cohort. Lastly, the scale was translated in English.

**Results:**

The systematic review included 137 articles. Focus groups (23 former patients and 5 relatives) highlighted communication challenges and the need for human support. Scale construction resulted in an initial 6-item pragmatic bedside tool. Face validation showed that the constructed scale was acceptable and led to refining the scale by changing item evaluation formats to a 4-stage Likert scale. Cohort validation, comprising 305 scale administrations (84 patients), indicated overall satisfaction and adequate completion rates. Psychometric analysis led to the removal of two items. A simplified 4-item scale (Comfort, Safety, Information, Trust) demonstrated improved reliability and coherence.

**Conclusion:**

The final scale provides a practical measure of patient well-being in ICUs and is usable in real time at the bedside.

**Supplementary Information:**

The online version contains supplementary material available at 10.1186/s13054-026-05923-z.

## Take home message

A simple, quick, and effective scale to assess, in real-time at the bedside, the well-being and sense of safety of intubated patients and all patients hospitalized in intensive care units, was co-constructed with former patients, relatives, and healthcare professionals.

**Supplementary Information:**

The online version contains supplementary material available at 10.1186/s13054-026-05923-z.

## Introduction

Critically ill patients admitted to intensive care units (ICU) undergo a highly stressful experience. Their short-term survival is at risk, and the environment is noisy, hostile, and overwhelming. The sense of well-being and security these patients feel in such an environment can have long-lasting effects. Many patients describe their ICU stay retrospectively as “a nightmare” or “a horror” [3–9], particularly due to communication difficulties and intense feelings of frustration [10, 11]. Intubated patients are especially vulnerable, as the tracheal tube significantly hinders communication by preventing vocalization and limiting the movement of the articulators needed for speech. Healthcare professionals are working to improve and humanize the ICU environment, a challenge identified in guidelines and placed on the intensive care community’s agenda for over a decade [12, 13]. It is now well recognized that an ICU stay can be traumatic, with long-lasting repercussions. After hospital discharge, patients and their relatives begin a long period of rehabilitation before being able to return to a “normal” life, with returning to work pose a major challenge [14].

Significant progress has been made, with multiple scales developed to evaluate communicative patients such as Visual analogical scale, but also non-communicative patients’ pain (e.g., Behaviour Pain Scale-BPS) [15], delirium (e.g., Confusion Assessment Method for the CAM-ICU) [16, 17], and agitation (e.g., Richmond Agitation Sedation Scale) [18].

Currently, to the best of our knowledge, no tool is available to assess the feeling of well-being and security of intubated patients in real time during their ICU stay. Measuring patient well-being and security while ventilated is essential for developing and evaluating initiatives aimed at improving patients’ quality of life in the ICU. Well-being and sense of security represent a short-term patient-centered outcome, complementing traditional outcomes such organ failure, mortality, and long-term quality of life assessed in intensive care trials. The sense of well-being and safety refers it an individual’s immediate sense of comfort and security (feeling physically and mentally at ease) and protected from harm or danger. This is a personal, emotional state that reflects how safe and content someone feels in their daily life. In contrast, quality of life is a broader and more comprehensive concept that includes many different aspects of life, such as health, income, education, environment, social relationships, and more. It encompasses both objective conditions and subjective perceptions. More specifically, health-related quality of life is a multidimensional construct encompassing physical, emotional, and social functioning, as well as individuals’ perceptions of their overall health status. While the sense of well-being and safety is important part of quality of life, the latter goes beyond just emotions to include various measurable factors affecting a person’s overall living conditions [19]. Building on this, wellness is conceptualized as a broad, dynamic, and holistic process involving active engagement in lifestyle choices across physical, psychological, and social dimensions [1, 2]. In contrast, well-being refers to a subjective state or outcome reflecting an individual’s experienced sense of balance and feeling well [1].

Our objective, after conduction a systematic review of outcomes used to evaluate well-being and security of intubated patients, was to develop a dedicate scale assessing “*the sense of well-being and security*” of these patients through a multi-professional co-construction process involving ICU patients and relatives.

## Materials and methods

### Study design

This study was conducted in two phases. The first phase consisted of constructing a scale to quantify patient’s feelings of well-being and security while in the ICU. Items were generated through a prior systematic review and transcripts of focus groups conducted with patients and relatives. The scale was constructed by a panel of patients and professional experts using a Delphi methodology. The second phase of the study consisted of validating the scale in four French ICUs through patient interviews and the follow-up of a cohort of eighty-four patients. Lastly, the scale was translated into English with back-translation and cross-validation.

The study protocol was approved by the regional ethics committee (Espace de reflexion éthique région Centre val de Loire, no.2010.094) in accordance with French law.

### First phase: step 1 -systematic review

The systematic review aimed to identify adult and pediatric studies with endpoints evaluating patients’ well-being while admitted to an ICU. The question of the systematic review was: “What are the endpoint used in all studies (interventional or not) to assess the well-being of patients (adults or pediatrics) hospitalized in intensive care?”. The PICOs question was: P (population) adults and children hospitalized in intensive care (neonatologies excluded), I (intervention) all the interventions (medicated or not), C (comparator) all the comparators, O (outcomes) all the outcomes linked to well-being and feeling of security for the patient and S (studies) all the studies. The three research equations were carried out with the help of a documentalist (see paragraph Research equation), in the database Medline, with a research end date of December 31, 2019. The articles included in the review were those published in English or French. No restriction was made regarding the type of studies selected (prospective, retrospective, interventional or not, randomized or not, and observational studies, including questionnaires, in Humans), were included in the review. The main objective was to collect the endpoint evaluating the feeling of well-being of the patient hospitalized in adult or pediatric intensive care unit (exclusion of neonatology). The non-inclusion criteria were the feeling of well-being of relatives, and studies concerns the end of life in intensive care.

The selection of studies on title and abstract was carried out by two independent reviewers (LBC and JC) and a third reviewer (SE) in the event of discordance. The selection of studies based on full text and data extraction were carried by two independent reviewers among five reviewers (AL, HM, MB, JC, LBC). In case of disagreement between the two reviewers, a third or even a fourth reviewer among the different reviewers was questioned.

Relevant endpoints retrieved from the systematic review were presented using word cloud to the scale construction committee. Methodological details are provided in the online Appendix. The review was prospectively registered in PROSPERO (CRD42020213581).

### First phase: step 2- exploratory inquiry, interviews and focus groups

A sociologist (HL) conducted focus groups in October, November, and December 2020 (through videoconferencing due to pandemic restrictions). Initial individual interviews with patients were conducted to prepare the focus group sessions and refine the questions. Four 2-hour focus groups were performed with former patients and relatives. The objective of recruiting patients and relatives was to conduct qualitative research that highlights the needs and difficulties of intubated patients from admission to discharge from the ICU. All patients included had been hospitalized in an ICU and had received invasive mechanical ventilation for at least 72 h. The relatives were eligible if they had a loved-one who met these criteria and had visited their relative in the unit regularly, including at least twice while the patient was intubated.

Focus groups addressed the major topics: What do former patients and close relatives ones say about this remote care experience? What difficulties did they encounter and what were their needs? What contributes to this feeling of security? How to preserve this feeling while providing the best possible support during discharge from the hospital The content of the focus group was analyzed from transcription scripts using a qualitative method implementing thematic analysis (searching for recurring and important themes for patients and their relatives, analyzing the words used and identifying recurring concepts) [20]. Before focusing on the well-being of patients with PTSD, the exploratory survey had to analyze what they needed to feel safer. A report was generated, including the major themes to be explored to consider the construction of the scale. A summary was transferred to the scale construction committee, along with the systematic review word cloud.

### First phase: step 3-scale construction committee

Healthcare professionals (nurses, auxiliary nurses, physicians, speech therapists, physiotherapists with at least two years of ICU experience), researchers, three former patients, and one relative participated in the multidisciplinary expert committee. Based on the focus groups transcripts and summary, as well as the systematic review word cloud, the committee generated an initial list of items. This list was then submitted to the global panel (all participants to the focus groups and committee members) to refine the scale through three rounds of Delphi methodology (see online Appendix). For each round of the Delphi process, each panelist received a link by email to access the questionnaire and respond independently, blinded to the answers of other participants. The committee produced the final list of items for the validation process.

### Second phase: scale validation

The validation phase consisted of individual patient interviews to assess face validity and, secondarily, the implementation of the scale in a cohort of ICU patients. Interviews were conducted for face validity: the scale was administered to intubated patients in a single ICU (Tours) prior to extubation, and the interviews took place within a few hours after extubation [21].

The cohort was recruited from four ICUs (Angers, Le Mans, Orléans, and Tours) between March 3rd, and August 31 st, 2022 (see online Appendix). Included patients were adults with a RASS score between − 1 and 0, who were not under legal protection, had provided informed consent to participate, and were fluent in French. “The scale was administered by the healthcare professionals in charge of the patient, without prior specific training, as all instructions for administering the scale were provided in the document.”

The analysis focused on evaluating the scale’s psychometric structure, including:

- confirmatory factor analysis (CFA) to identify items potentially misclassified within their designated dimensions;
- internal consistency of each dimension using Cronbach’s alpha (expected value greater than 0.7) [22];
- scalability using Loevinger’s H index (expected value greater than 0.3) [23].

CFA model fit was assessed using the Confirmatory Fit Index (CFI – expected values greater than 0.9), Tucker-Lewis Index (TLI - expected values greater than 0.9) and Root Mean Square Error of Approximation (RSMEA – expected value lesser than 0.08).

Items presenting psychometric issues-missclassification (i.e., items presenting stronger factor loading on another dimension than the one in which they were classified), low factor loading (< 0.4), generating a decrease in internal consistency, or with poor scalability (H < 0.3)) were discussed by the committee, and a final French version of the scale was establish by consensus.

User satisfaction (patients and healthcare professionals) was evaluated using a 4-point Likert scale.

The final scale was translated into English and independently back-translated to French by professional translators and cross-validated with the initial version [24], with the aim of enabling publication in an English-language journal.

## Results

### Systematic review

**Fig. 1:**
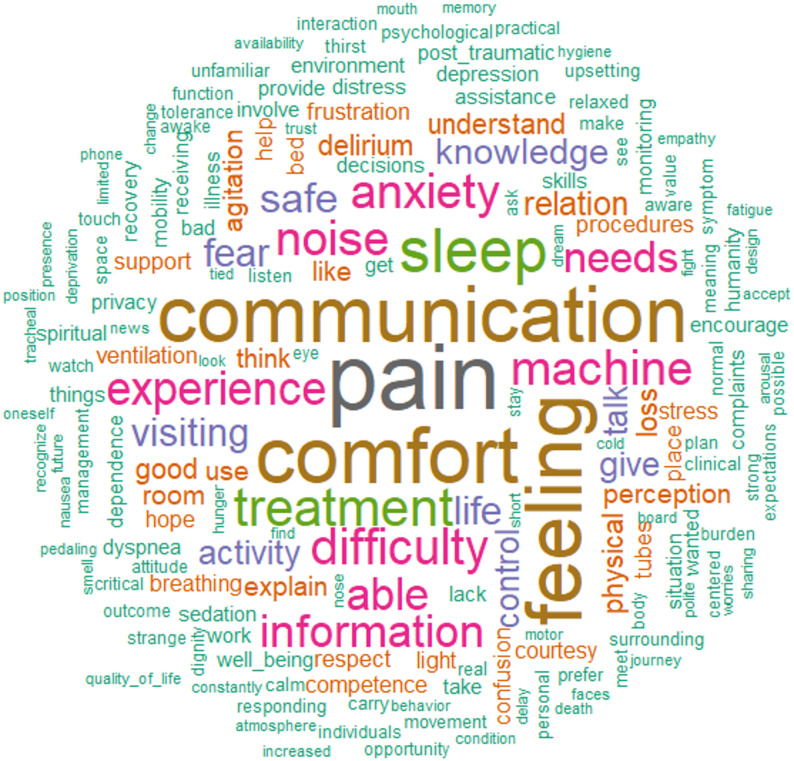
Systematic review results presented as a word cloud to experts

Among 883 articles initially retrieved, 137 (15%) were finally included (see online Appendix for the study selection and article characteristics, and e-Table 1, e-Table 2, e-Figure 1). The final word cloud of items evaluating patients’ well-being while admitted to an ICU among the included studies is presented in Fig. 1.

### Focus group

Five initial individual interviews with patients were conducted to prepare the focus group sessions. Twenty-three former patients and five relatives (average age: 54 years), including 13 women and 15 men, participated (see online Appendix for details, and e-Table 3). The two main themes of concern and interest identified were: 1) challenges related to communication and information throughout all stages of care – upon arrival, during hospitalization, and at discharge from the unit. During hospitalization, there are insufficient tools for communicating with intubated patients. Considering other means of communication, particularly those involving family members, would reduce stress levels and give patients a more active role in their care.

2) the need for presence and human support to promote patient-centered care. Everything that is already being done by paramedical healthcare professionals and that falls under the heading of “humanizing care” should be continued and encouraged. These are everyday actions that allow people to feel respected and maintain a certain dignity, despite their state of dependence.

Our results also emphasize the importance of talking to sick people and their loved ones on a daily basis, whether or not the patient is able to communicate, in order to reduce stress and prevent feelings of persecution from worsening in some individuals. Key themes for consideration of the scale are presented in the online Appendix.

### Scale construction

The expert panel was composed of 23 healthcare professionals, 6 researchers, and 35 patients and relatives. Details are provided in the online Appendix. Initial multidisciplinary expert committee discussions highlighted the need for a pragmatic scale, usable at the patient’s bedside. As patients are often very tired, the scale must be simple and easy to complete. Since healthcare professionals are primarily engaged in technical tasks, the scale needs to be practical and not too time-consuming. The importance of using positive wording in the scale items was identified. It was decided to develop a scale to be used during the ICU stay, including admission but excluding discharge and follow-up beyond the ICU, despite this topic having emerged from the focus group.

The details of the Delphi consensus process, covering the target population, scale dimensions and items, scale size, response modality (yes/no versus quantification), and ideal frequency of scale administration to patients, are detailed in the online Appendix (e-Table 4).

The Delphi process led to the following conclusions: the scale must be usable for all ICU patients and adapted to intubated patients. The dimensions retained were sense of physical security, emotional well-being, feeling of anxiety, feeling of emotional security, and relational well-being. The items retained were: “I feel comfortable”, “I feel safe”, “I feel well informed”, “I feel considered as a person”, “I feel anxious”, and “I feel confident”. The frequency of administration should be at least twice a day for intubated patients.

Ongoing committee discussions on the answer format (Yes/No versus numerical visual scale versus Likert scale versus combination of these formats) led the committee to decide to test these answer formats in real-life during the first phase of validation.

### Scale face validation

Eleven individual interviews were carried out for face validity. The scale was administered to five women and eight men, aged 43 to 71 years. One patient was not extubated after administration of the scale, and three patients could not answer the post-extubation questionnaires due to severe confusion. The scale was administered with the “Yes/No” modality and then a numerical rating if answered “yes” to quantify a specific item. It was decided, with patient input, to modify the quantification to a Likert scale comprising “not at all”, “a little”, “well”, and “very well”. Patients evaluated the scale as appropriate and feasible in terms of lengths and effort required to respond.

The 6-item scale used for the next step is presented in the online Appendix. The scale ranged from 0 to 18: 0 representing a very negative feeling and 18 a very positive feeling for the patient.

### Scale cohort validation

Eighty-four adults patients were included (average age 65 years, Simplified Acute Physiology Score (SAPS)II 46±18, 66 (79%) intubated), and a total of 305 scale administrations were evaluated. Detailed patients’ characteristics are provided in the online Appendix (e-Table 5 to e-Table 10).

The scale was generally well received: 220 (89%) patients reported being satisfied or very satisfied with the instrument, 266 (87%) healthcare professionals considered the scale administration time appropriate, and 220 (72%) expressed satisfaction of high satisfaction with the scale.

The main problem with the scale implementation was that in 39 cases (13%), scale administration failed (fatigued patient who falls asleep while the questions are being asked, patient who does not appear to understand). Of the 305 performed scale administrations, 284 were complete.

### Psychometric characteristics of the initial 6-item scale

Values scores ranged from 0 to 18 points, with a mean of 12.2 (± 3.1) and a median of 12 (minimum 0, maximum 18). The distribution was uniform, with no notable ceiling or floor effects and no substantial asymmetry (online Appendix, e-Figs. 2 and e and 3).

Multiple psychometric indicators identified item 5 (anxiety assessment) as problematic, suggesting that it did not align with the unidimensional structure measured by the other items. Notably, this item was also the only negatively worded item.

Based on these findings, the expert committee decided to remove anxiety assessment item to improve scale’s overall psychometric performance.

### Simplified 5-item scale psychometric evaluation

The total score for the five-items scale ranged from 0 to 15: 0 indicating a very negative perception and 15 a very positive one for the patient. Score was distributed from 0 to 15 points, with a mean of 10.8 (± 2.9) and a median of 11. A slight ceiling effect was observed (12% of individuals reached the maximum score, see online Appendix, e-Figs. 4 and e and 5).

All items demonstrated good internal coherence. However, achieving an adequate model fit required specifying a correlation between the residuals of two items (“well informed” and “considered as a person”), suggesting that these items were more strongly related than expected given the overall item structure. To avoid redundancy and improve factorial clarity, the committee decided to remove the item “considered as a person”.

### Final simplified 4-item scale psychometric evaluation

The scale fit was good (CFI > 0.99, TLI > 0.99, and RMSEA = 0.02). Although the scale lost a little reliability, the alpha of 0.83 remained good, especially for a scale with only four items.

The expert panel concluded on the simplest possible scale to minimize patient fatigue. Following the presentation of the statistical analyses, the experts agreed to retain the four proposed items.

An updated review through December 31, 2023, found no articles that could have altered the expert discussions.

**Fig. 2:**
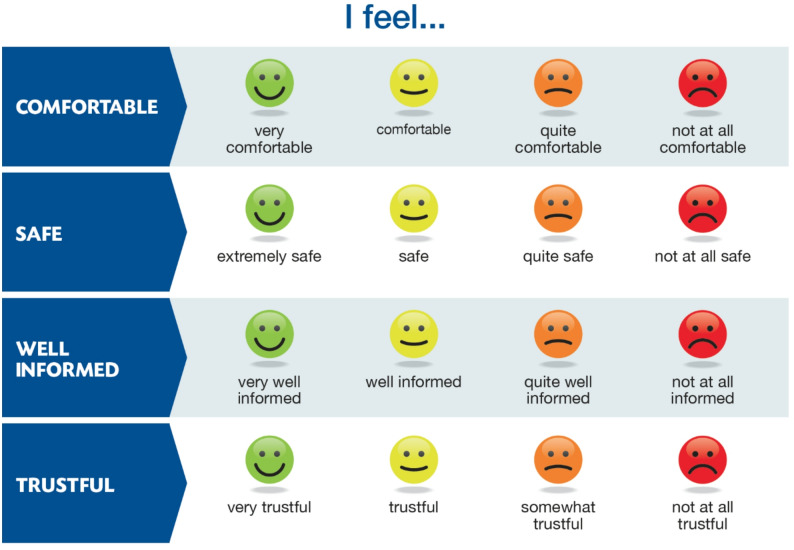
TOURS Scale: PRACTICAL COCONSTRUCTED INTENSIVE CARE PATIENTS’ WELL BEING AND SAFETY SCALE Dear Sir or Madam, we are going to show you four sentences regarding how you currently feel. There are four possible answers for each sentence. I will read each sentence out to you with the possible answers one by one. Then I will read each of the options again so that you can give me your answer by either moving your hand or your head

The final scale was translated into English and independently back-translated into French without noticeable alterations. The final English wording of the scale is presented in Fig. 2.

## Discussion

This study successfully developed and validated the first scale specifically designed to evaluate the well-being and sense of security of intubated adult patients in ICUs. The scale was created through a multi-professional, co-constructed approach that incorporated the perspectives of patients, relatives, and healthcare providers. It can be used to evaluate actions aimed at improving patient care and humanizing intensive care unit [3, 25, 26].

The need for a tool to measure the well-being of intubated patients is critical, as this patient group faces significant communication barriers, making them particularly vulnerable. ICU patients commonly experience feelings of distress, anxiety, and helplessness due to the inability to communicate effectively while intubated [27–30]. This is especially significant given the evidence that poor communication in the ICU is associated with increased levels of anxiety, depression, and posttraumatic stress disorder (PTSD) after discharge. The scale developed in this study aligns with these concerns, emphasizing the importance of creating opportunities for patients to express their needs and feel understood, which might mitigate feelings of powerlessness and improve their overall ICU experience [31]. An early communication strategy focused on the patient’s needs and feelings helps to improve respect for the patient dignity [32–34]. Within this framework, nursing care consistently emerges as a central factor, with intensive care patients frequently identifying it as a critical component influencing their perception of safety during hospitalization [35].

Our results indicate that the scale, which includes items such as “I feel comfortable” and “I feel well-informed,” addresses critical aspects of patient well-being that are often overlooked in traditional assessments. Several studies have pointed out that intubated patients frequently report feeling disoriented, anxious, and uninformed about their treatment [3–5]. These feelings of uncertainty can exacerbate their emotional distress, making it even more difficult for them to process their medical situation and contribute to decisions about their care. By focusing on dimensions such as emotional well-being, physical security, and relational well-being, this scale complements traditional clinical outcomes, offering a more holistic view of the patient’s ICU experience. This approach is consistent with contemporary shifts toward person-centered care, where the emotional and psychological states of patients are considered integral to their overall health and recovery [32].

The process of scale development aimed to adhere to best practices in health research. The Delphi technique is widely used to establish consensus on complex issues, particularly in fields with limited existing measurement tools [36]. In this study, the involvement of both healthcare professionals and former patients ensured that the scale was clinically relevant while also reflecting patient experiences. This approach enhances the tool’s validity by incorporating diverse perspectives and minimizing potential bias in item selection.

However, the scale’s effectiveness depends on its practicality in clinical settings. Given that ICU staff often work under significant time constraints, the scale was designed to be brief and easy to use, ensuring it can be administered efficiently without overwhelming patients or healthcare professionals [37, 38].

This study has some limitations. Despite the delay between completing the systematic review and finalizing the scale due to pandemic-related challenges, the study’s rigor was preserved. While the systematic review provided a comprehensive background, it was conducted using only one database, which may have restricted the scope of included studies. Moreover, although the focus group methodology was valuable, it could introduce retrospective bias, as participants reflected on their experiences after ICU discharge. Future studies should aim to include a broader sample of ICU patients and relatives and explore the scale’s applicability across different types of ICUs. Furthermore, the construction committee decided to remove the item “considered as a person” rather than “well informed.” While this decision was reached through consensus and extensive discussion, it may still be considered subjective. Retaining “considered as a person” instead of “well informed” might have led to different results. Although adding a covariance variable between the residuals associated with these two items was technically feasible, it would have resulted in a more complex scale, potentially reducing homoscedasticity. Additionally, while the scale is intended to complement other tools assessing pain, agitation, and delirium during the ICU stay, as well as discomfort at ICU discharge, these complementary scales were not used in the present validation cohort (12–17). The validation was based on 305 evaluations, but with a limited sample size of 84 patients, which could impact the generalizability of the findings. Future studies should aim to validate the scale in a more diverse patient population, ensuring its robustness and applicability across different healthcare settings, while also comparing it with other complementary ICU assessment tools. The validation step on an English-speaking sample with psychometric analysis was not carried out. This provide an interesting English version to validate among a sample of English-speaking patients to meet current guidelines [24]. Moreover, additional studies are required to assess the association of this scale with other intensive care parameters, in particular delirium measured using the CAM-ICU. Finally, real-life implementation studies should be planned to assess the feasibility and practical use of the scale in routine ICU settings, and to evaluate its validity for repeated measurements in long-stay patients. Further research is also needed to evaluate the long-term benefits of using this scale, particularly it potential impact on post-ICU outcomes such as psychological distress (e.g., PTSD) and quality of life.

## Conclusion

This study introduces a novel and valuable tool for assessing the well-being and sense of security of intubated adult patients in the ICU. The scale is easy to use, consisting of only four items, and could be implemented to enhance the quality of care by enabling healthcare providers to monitor and address patients’ emotional and psychological needs alongside their physical health. Given the increasing recognition of the psychological impact of critical illness and ICU admission, this scale could play a significant role in improving the overall patient experience in these high-stress environments.

## Supplementary Information

Supplementary Material 1

## Data Availability

The dataset used in this study is available from the corresponding author on reasonable request.

## References

[1] Eriksson M, Ekström-Bergström A, Arvidsson S, Jormfeldt H, Thorstensson S, Åström U, et al. Meaning of wellness in caring science based on Rodgers’s evolutionary concept analysis. Scand J Caring Sci. 2024;38:185–99. 10.1111/scs.13196. 37507842 10.1111/scs.13196

[2] Skarbek A, Endsley P, Chrisman MS, Hastert M, Stellwagen C. Exploring wellness through concept analysis. J Sch Nurs Off Publ Natl Assoc Sch Nurses. 2024;40:86–96. 10.1177/10598405231165510. 10.1177/10598405231165510 37070135

[3] Kvande ME, Angel S, Højager Nielsen A. Humanizing intensive care: a scoping review (HumanIC). Nurs Ethics. 2022;29:498–510. 10.1177/09697330211050998. 34894870 10.1177/09697330211050998 PMC8958643

[4] Nielsen AH, Kvande ME, Angel S. Humanizing and dehumanizing intensive care: thematic synthesis (HumanIC). J Adv Nurs. 2023;79:385–401. 10.1111/jan.15477. 36281216 10.1111/jan.15477 PMC10092106

[5] Bohart S, Lamprecht C, Andreasen AS, Waldau T, Møller AM, Thomsen T. Perspectives and wishes for patient and family centred care as expressed by adult intensive care survivors and family-members: a qualitative interview study. Intensive Crit Care Nurs. 2023;75:103346. 10.1016/j.iccn.2022.103346. 36470701 10.1016/j.iccn.2022.103346

[6] Zetterlund P, Plos K, Bergbom I, Ringdal M. Memories from intensive care unit persist for several years–a longitudinal prospective multi-centre study. Intensive Crit Care Nurs. 2012;28:159–67. 10.1016/j.iccn.2011.11.010. 22579396 10.1016/j.iccn.2011.11.010

[7] Jones C, Griffiths RD, Humphris G, Skirrow PM. Memory, delusions, and the development of acute posttraumatic stress disorder-related symptoms after intensive care. Crit Care Med. 2001;29:573–80. 10.1097/00003246-200103000-00019 11373423 10.1097/00003246-200103000-00019

[8] Cazorla C, Cravoisy A, Gibot S, Nace L, Levy B, Bollaert P-E. [Patients’ perception of their experience in the intensive care unit]. Presse Médicale Paris Fr 1983. 2007;36:211–6. 10.1016/j.lpm.2006.07.008. 10.1016/j.lpm.2006.07.008 17259029

[9] Simini B. Patients’ perceptions of intensive care. Lancet. 1999;354:571–2. 10.1016/S0140-6736(99)02728-2. 10470711 10.1016/S0140-6736(99)02728-2

[10] Tembo AC, Higgins I, Parker V. The experience of communication difficulties in critically ill patients in and beyond intensive care: findings from a larger phenomenological study. Intensive Crit Care Nurs. 2015;31:171–8. 10.1016/j.iccn.2014.10.004. 25486970 10.1016/j.iccn.2014.10.004

[11] Carroll SM. Nonvocal ventilated patients perceptions of being understood. West J Nurs Res. 2004;26(1):85–103. 10.1177/0193945903259462. 14984652 10.1177/0193945903259462

[12] SFAR, SRLF A. Conference de Consensus Mieux Vivre la Réanimation. 2009 [cited 2024 Dec 8]; https://www.srlf.org/wp-content/uploads/2015/12/2009_11_19_conference_de_consensus_commune_mieux_vivre_en_reanimation.pdf. Accessed 8 Dec 2024

[13] Devlin JW, Skrobik Y, Gélinas C, Needham DM, Slooter AJC, Pandharipande PP, et al. Clinical Practice Guidelines for the Prevention and Management of Pain, Agitation/Sedation, Delirium, Immobility, and Sleep Disruption in Adult Patients in the ICU. Crit Care Med. 2018;46:e825–73. 10.1097/CCM.0000000000003299 10.1097/CCM.0000000000003299 30113379

[14] Granja C, Amaro A, Dias C, Costa-Pereira A. Outcome of ICU survivors: a comprehensive review. The role of patient-reported outcome studies. Acta Anaesthesiol Scand. 2012;56:1092–103. 10.1111/j.1399-6576.2012.02686.x. 22471617 10.1111/j.1399-6576.2012.02686.x

[15] Aïssaoui Y, Zeggwagh AA, Zekraoui A, Abidi K, Abouqal R. Validation of a behavioral pain scale in critically ill, sedated, and mechanically ventilated patients. Anesth Analg. 2005;101:1470–6. 10.1213/01.ANE.0000182331.68722.FF. 16244013 10.1213/01.ANE.0000182331.68722.FF

[16] Ely EW, Inouye SK, Bernard GR, Gordon S, Francis J, May L, et al. Delirium in mechanically ventilated patients: validity and reliability of the confusion assessment method for the intensive care unit (CAM-ICU). JAMA. 2001;286:2703–10. 10.1001/jama.286.21.2703 11730446

[17] Ely EW, Margolin R, Francis J, May L, Truman B, Dittus R, et al. Evaluation of delirium in critically ill patients: validation of the Confusion Assessment Method for the Intensive Care Unit (CAM-ICU). Crit Care Med. 2001;29:1370–9. 10.1001/jama.286.21.2703 10.1097/00003246-200107000-00012 11445689

[18] Sessler CN, Gosnell MS, Grap MJ, Brophy GM, O’Neal PV, Keane KA, et al. The Richmond Agitation-Sedation Scale: validity and reliability in adult intensive care unit patients. Am J Respir Crit Care Med. 2002;166:1338–44. 10.1164/rccm.2107138 10.1164/rccm.2107138 12421743

[19] Pinto S, Fumincelli L, Mazzo A, Caldeira S, Martins JC. Comfort, well-being and quality of life: discussion of the differences and similarities among the concepts. Porto Biomed J. 2017;2:6–12. 10.1016/j.pbj.2016.11.003. 32258577 10.1016/j.pbj.2016.11.003 PMC6806988

[20] Green J, Thorogood N. Qualitative Methods for Health Research. SAGE; 2018.

[21] Terwee CB, Prinsen C, Chiarotto A, Westerman MJ, Patrick DL, Alonso J, et al. COSMIN methodology for evaluating the content validity of patient-reported outcome measures: a Delphi study. Qual Life Res. 2018;27:1159–70. 10.1007/s11136-018-1829-0. 29550964 10.1007/s11136-018-1829-0 PMC5891557

[22] Cronbach L. Coefficient alpha and the internal structure of tests. Psychometrika. 1951;16:297–333.

[23] Loevinger J. The technic of homogeneous tests compared with some aspects of scale analysis and factor analysis. Psychol Bull. 1948;45:507–29. 18893224 10.1037/h0055827

[24] Beaton DE, Bombardier C, Guillemin F, Ferraz MB. Guidelines for the process of cross-cultural adaptation of self-report measures. Spine. 2000;25:3186–91. 10.1097/00007632-200012150-00014. 11124735 10.1097/00007632-200012150-00014

[25] Cheraghi MA, Esmaeili M, Salsali M. Seeking humanizing care in patient-centered care process: a grounded theory study. Holist Nurs Pract. 2017;31:359–68. 10.1097/HNP.0000000000000233. 29028774 10.1097/HNP.0000000000000233

[26] Velasco Bueno JM, La Calle GH. Humanizing intensive care: from theory to practice. Crit Care Nurs Clin North Am. 2020;32:135–47. 10.1016/j.cnc.2020.02.001. 32402312 10.1016/j.cnc.2020.02.001

[27] Ten Hoorn S, Elbers PW, Girbes AR, Tuinman PR. Communicating with conscious and mechanically ventilated critically ill patients: a systematic review. Crit Care. 2016;20:333. 10.1186/s13054-016-1483-2. 27756433 10.1186/s13054-016-1483-2 PMC5070186

[28] Bodet-Contentin L, Gadrez P, Ehrmann S. Eye-tracking and speech-generating technology to improve communication with intubated intensive care unit patients: initial experience. Intensive Care Med. 2018. 10.1007/s00134-018-5093-0. 29502253 10.1007/s00134-018-5093-0

[29] Bodet-Contentin L, Szymkowicz E, Delpierre E, Chartier D, Gadrez P, Muller G, et al. Eye tracking communication with intubated critically ill patients: a proof-of-concept multicenter pilot study. Minerva Anestesiol. 2022;88:690–7. 10.23736/S0375-9393.22.16275-9. 35546732 10.23736/S0375-9393.22.16275-9

[30] Guttormson JL, Bremer KL, Jones RM. Not being able to talk was horrid”: a descriptive, correlational study of communication during mechanical ventilation. Intensive Crit Care Nurs. 2015;31:179–86. 10.1016/j.iccn.2014.10.007. 25579081 10.1016/j.iccn.2014.10.007 PMC4466051

[31] Freeman-Sanderson A, Brodsky MB, Dale C, Gupta A, Haines K, Happ MB, et al. A Core Outcome Set for Research Evaluating Interventions to Enable Communication in Patients With an Artificial Airway: An International Delphi Consensus Study (Comm-COS). Crit Care Med. 2024;52:e450–62. 10.1097/CCM.0000000000006347 10.1097/CCM.0000000000006347 PMC11321605 38899947

[32] Halvorsen K, Jensen JF, Collet MO, Olausson S, Lindahl B, Saetre Hansen B, et al. Patients’ experiences of well-being when being cared for in the intensive care unit-an integrative review. J Clin Nurs. 2022;31:3–19. 10.1111/jocn.15910. 34159663 10.1111/jocn.15910

[33] Al-Shamaly HS. Patterns of communicating care and caring in the intensive care unit. Nurs Open. 2022;9:277–98. 10.1002/nop2.1061. 34536338 10.1002/nop2.1061 PMC8685886

[34] Moen EK, Nåden D. Intensive care patients’ perceptions of how their dignity is maintained: a phenomenological study. Intensive Crit Care Nurs. 2015;31:285–93. 10.1016/j.iccn.2015.03.003. 25963294 10.1016/j.iccn.2015.03.003

[35] Wassenaar A, Schouten J, Schoonhoven L. Factors promoting intensive care patients’ perception of feeling safe: a systematic review. Int J Nurs Stud. 2014;51:261–73. 10.1016/j.ijnurstu.2013.07.003. 23910399 10.1016/j.ijnurstu.2013.07.003

[36] Diamond IR, Grant RC, Feldman BM, Pencharz PB, Ling SC, Moore AM, et al. Defining consensus: a systematic review recommends methodologic criteria for reporting of Delphi studies. J Clin Epidemiol. 2014;67:401–9. 10.1016/j.jclinepi.2013.12.002. 24581294 10.1016/j.jclinepi.2013.12.002

[37] Dauvergne JE, Bruyneel A, Caillet A, Caillet P, Keriven-Dessomme B, Tack J, et al. Workload assessment using the nursing activities score in intensive care units: nationwide prospective observational study in France. Intensive Crit Care Nurs. 2025;87:103866. 10.1016/j.iccn.2024.103866. 39482222 10.1016/j.iccn.2024.103866

[38] Caillet A, Dauvergne JE, Poiroux L, Blanchard P-Y, Bruyneel A. Analysis of the prevalence of missed nursing care using three workload assessment methods: a nationwide cross-sectional study among intensive care nurses. Nurs Crit Care. 2025;30:e70055. 10.1111/nicc.70055. 40384582 10.1111/nicc.70055

