# Supplementary material for: Well-being and sense of security of intubated patients in intensive care units: a patient co-constructed dedicated scale
Source: Crit Care. 2026 Mar 7;30:113. doi: 10.1186/s13054-026-05923-z (PMC12983626; doi:10.1186/s13054-026-05923-z)
Supplement: Supplementary file 1 — Supplementary Material 1 [file 13054_2026_5923_MOESM1_ESM.docx]

**Well-being and security feeling of intubated patient in intensive care units: a patient co-constructed dedicated scale**

Laetitia Bodet-Contentin, Hélène Lecompte, Adrien Lociciro, Nancy Kentish Barnes, Hélène Messet, Misylias Bouaoud, Justine Cibron, Nicolas Chudeau, François Barbier, Carole Haubertin, Laurent Poiroux, Benedicte Sautenet, Wissam El Hage, Julie Leger, Amélie Le Gouge, Jean-Benoit Hardouin, Stephan Ehrmann

| **Online Appendix**  **Systematic review**  Methods of the systematic review  The data extraction was as follows: first author, publication date, name of the revue, first endpoint, secondary endpoints, study type (interventional, randomized, blinded, cross over, cohort, questionnaires, observational), if interventional the type of intervention, number of participants, population type (adult, children), well-being endpoint with its name, definition and methods of collection and evaluation.  As this is a qualitative systematic review, an assessment of risk of bias would not be appropriate. Studies were selected on their relevance to the research question.  We provided a narrative summary of the included studies. We presented the results with a word cloud with the software R (Fellows I (2018). _wordcloud: Word Clouds_. R package version 2.6, <https://CRAN.R-project.org/package=wordcloud>). R Core Team (2024). _R: A Language and Environment for Statistical Computing_. R Foundation for Statistical Computing, Vienna, Austria. <https://www.R-project.org/>  We have updated the review until December 31, 2023.   \| *Research equation of the review*  #1,"Search (care, comfort[MeSH Terms]) AND ((((critical care[MeSH Terms]) OR critical care nursing[MeSH Terms]) OR intensive care units[MeSH Terms]) OR critical illness[MeSH Terms])" \| \| --- \| \| #2,"Search (feeling safe) AND ((((critical care[MeSH Terms]) OR critical care nursing[MeSH Terms]) OR intensive care units[MeSH Terms]) OR critical illness[MeSH Terms])"  #3,"Search (((((critical care[MeSH Terms]) OR critical care nursing[MeSH Terms]) OR intensive care units[MeSH Terms]) OR critical illness[MeSH Terms])) AND ((((patient preference[MeSH Terms]) OR patient satisfaction[MeSH Terms]) OR patient comfort[MeSH Terms]))" \| |
| --- | --- | --- |

Results of the systematic review

The three search equations resulted in finding 883 articles. Of these articles, 48 were duplicates. 835 articles have been selected. Among these articles, we kept 275 after having resolved 188 discrepancies, after reading the title and abstract. Of these 275 articles, 233 full texts were obtained. Among these full texts, we have selected 178, after having resolved 90 discrepancies. 137 (58%) had at least one endpoint concerning well-being in intensive care. Articles’ characteristics are presented in e-Table 1. Forty-nine articles (38%) were multicenter study. For the 113 articles that included patients, the median was 72 (28-213) patients with a minimum of 1 and a maximum of 2105. Among the interventional studies, the intervention concerned comfort and/or communication for 17 articles (53%) and technical care for 11 articles (37%). The country of origin of the articles selected in the systematic review is indicated in e-Table 2.

All the words entered in the data collection on the title and the definition of the endpoint were used to create the word cloud. Then we organized the words according to their thematic categorization. We deleted those that didn’t appear irrelevant to us, such as units of time, for example. Thus, we obtained a more relevant and easier word cloud 2. The terms “patient”, “relatives” and “caregiver” are about the ICU “actors”. “satisfaction” is a general term. We deleted them. And finally, we deleted the term “care” because it is the goal of hospitalization and we obtained the definitive word cloud (Figure 1). The construction of the word cloud step by step was presented in eFigure 1.

The update of the review resulted in 146 articles. Of these articles, 24 were duplicates. 32 articles have been selected. 28 full texts were obtained. Among these full texts, we have selected 21 full texts. The terms were from the same themes, such as pain, anxiety, comfort, and stress.

e-Table 1: Characteristics of the 137 articles of the systematic review having at least one endpoint related to the feeling of well-being of the patient hospitalized in ICU

| Year of publication |  |  |
| --- | --- | --- |
|  | < 2000 | 11 (8%) |
|  | 2000-2010 | 35 (26%) |
|  | 2011-2019 | 91 (66%) |
| Population |  |  |
|  | Adult | 129 (94%) |
|  | Pediatric | 5 (4%) |
|  | Mixed | 3 (52%) |
| Studies type |  |  |
|  | Editorial, expert opinion | 13 (9%) |
|  | Randomized trial | 19 (14%) |
|  | Non-randomized interventional trial | 19 (14%) |
|  | Observational study | 43 (31%) |
|  | Qualitative research | 30 (21%) |
|  | Literature review | 13 (9%) |

e-Table 2: Country of origin of articles selected in the systematic review

| **Country** |  |
| --- | --- |
| AUSTRALIA | 8 (6%) |
| BELGIUM | 1 (<1%) |
| BRAZIL | 3 (2%) |
| CANADA | 3 (2%) |
| CHINA | 2 (1%) |
| DENMARK | 1 (<1%) |
| ESTONIA | 1 (<1%) |
| EUROPE | 3 (2%) |
| FINLAND | 2 (1%) |
| FRANCE | 8 (6%) |
| GREECE | 1 (<1%) |
| INTERNATIONAL | 1 (<1%) |
| IRELAND | 2 (1%) |
| ITALY | 5 (4%) |
| JORDAN | 4 (3%) |
| KOREA | 1 (<1%) |
| LEBANON | 1 (<1%) |
| NETHERLANDS | 5 (4%) |
| NORWAY | 5 (4%) |
| PORTUGAL | 1 (<1%) |
| SINGAPORE | 1 (<1%) |
| SPAIN | 6 (4%) |
| SRI LANKA | 1 (<1%) |
| SWEDEN | 6 (4%) |
| SWITZERLAND | 1 (<1%) |
| TAIWAN | 2 (1%) |
| THAILAND | 2 (1%) |
| TURKEY | 7 (5%) |
| UK | 5 (4%) |
| USA | 48 (35%) |

**e-Figure 1**: Construction of the word cloud step by step


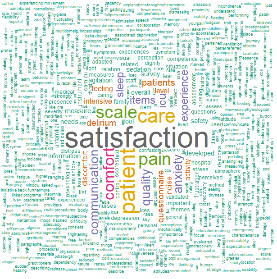


Word cloud with all the words collected with « endpoints name » and « definition of the endpoint »


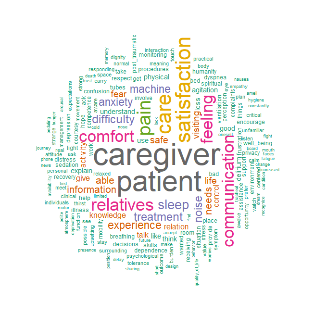


Word cloud with all the words collected organized according thematic categorization


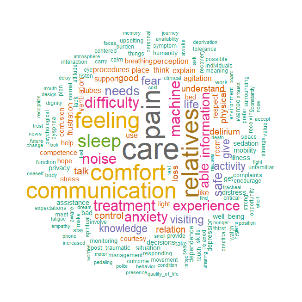


Word cloud after we deleted the words « patient » « relatives » « caregiver » and « satisfaction »


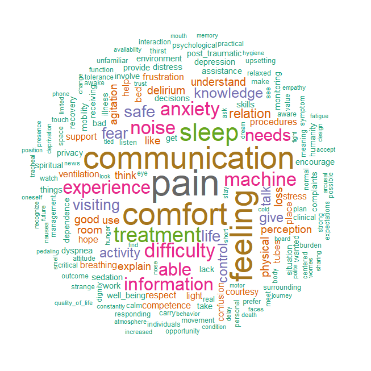


Word cloud after we deleted the term « care »

**Focus Groups**

Each ICU center contacted former patients who agreed to participate in the focus group, and then the sociologist reached out to them. Some declined to participate. The reason for refusal is indicated in the e-Table 3.

| e-Table 3: Reasons for refusal to participate to focus group   \| ICU \| Participation agreement \| Refusal to participate \| Lack of memory of their intensive care unit hospitalization \| Lack of computer equipment \| No answer to the phone call \| \| --- \| --- \| --- \| --- \| --- \| --- \| \| Orléans \| 8 \| 13 \| 0 \| 6 \| 23 \| \| Le Mans \| 10 \| 1 \| 0 \| 0 \| 2 \| \| Angers \| 8 \| 1 \| 0 \| 3 \| 15 \| \| Tours \| 17 \| 4 \| 2 \| 3 \| 9 \|   *Summary of the focus group analysis*  The two main themes of concern and interest identified were (1) the challenges of communication and information at all stages of care: upon arrival, during hospitalization, and at discharge from the service, and (2) the needs identified in terms of presence and human support to humanize care. The needs of communication theme comprised different aspects: lack of information provided by physicians, insufficient tools for communicating with intubated patients, and information deficit at discharge. The care process, described as violent despite goodwill of the teams, is always underscored by the patients. It puts the patient in powerlessness and dependence at all levels. Allowing patients to communicate would both reduce the stress felt and give them back a place as an actor in the care process. The needs identified within the theme of presence and human support were: the constant presence of relatives, which provides the patient with a sense of security and serves as a bridge between the patient and the healthcare professionals; improved communication, clear reference points, and the patient’s reappropriation of their experience despite limited or absent memories. It also appeared necessary to carry out “humanization of care” with daily attention: daily gestures that allow people to feel respected and maintained in dignity, despite their state of dependence. Another need identified was healthcare team speaking to patients and their relatives daily, whether the patient can communicate, to reduce or avoid the feeling of stress and persecution. In conclusion, the feeling of security was a central issue in ICU. It appeared important to inform the patient and to create spaces where they can be actors of their care to limit the feeling of dispossession and powerlessness throughout hospitalization.  *Themes for the scale proposed by the sociologist*  For each item, check: completely, most of the time, sometimes, don’t know.   - Relationship with the team - Sensations/emotions/perceptions (regarding oneself) - Feelings and needs (regarding the team and loved ones) - Projection into the future: sensations/emotions/needs - Scale construction committee and Delphi process   The characteristics of the committee participants and Delphi participants are presented below.  *Committee Participants:*   - 20 healthcare professionals: 2 intensivists, 11 nurses, 2 auxiliary nurses, one physiotherapist, one psychologist, one speech therapist and 2 nurses’ managers. Note that 2 healthcare professionals had a personal history of hospitalization in intensive care of which one had a personal history of intubation, 6 healthcare professionals had a relative who was hospitalized in intensive care - 6 researchers: 2 sociologists, 2 biostatistician-methodologists, psychiatrist, epidemiologist-nephrologist - 3 former patients: 2 men and one woman - 1 relative of a former patient (a men)   *Delphi Participants:*   - 30 participants of the Committee of experts - 3 other healthcare professionals: 3 intensivists (one woman and two men) - 31 former patients and relatives   The committee met in April 2021, 2nd and May 2021, 31. In the meantime, the Delphi consensus has been realized. For the first round, 55 (86%) participants completed the questionnaire. For the second round, 54 (98%) participants among the 55 who had responded to the first questionnaire completed the second questionnaire. For the third round, 52 (95%) participants among the 55 who had responded to the first questionnaire completed the third questionnaire.  *Delphi methodology*  The DELPHI method was carried out using questionnaires administered to experts and patients from the focus groups. The aim was to quantify (on a numerical scale from 1 “strongly disagree” to 9 “strongly agree”) the relevance and formulation of each item, along with an open-ended question on how to rephrase each item. Consensus was defined to retain an item if at least three-quarters of the respondents rate the relevance of an item at 7 or higher. Consensus for removing an item was defined if more than half of the respondents rate the relevance of an item at less than 5. For items that are neither retained nor removed, the expert group proposed a new formulation, and these items will be re-evaluated in the same way. This process continued for a maximum of three evaluations. For items that do not reach consensus after the third evaluation, the expert group made the final decision.  *Delphi results*  The Delphi results are presented in e-Table 4. |
| --- | --- | --- | --- | --- | --- | --- | --- | --- | --- | --- | --- | --- | --- | --- | --- | --- | --- | --- | --- | --- | --- | --- | --- | --- | --- | --- | --- | --- | --- | --- |

| eTable 4: Delphi results | | | | | |  |
| --- | --- | --- | --- | --- | --- | --- |
| First round | | | Second round |  | | Third round |
| Propositions | Results | | Proposition | Results | | Ranking of scale item importance (1=Highest, 9=Lowest) |
| **Population** | | | | | |  |
| ICU patients able to open and close their eyes on demand | 46% Agree | Expert committee discussed the propositions and consensually proposed a new formulation | The scale must be usable among all ICU patients and suitable for intubated patients | Validated (100% Agree) | |  |
| Intubated and ventilated ICU patients able to open and close their eyes on demand | 55% Agree |  |  |  |  |  |
| **Scale Dimensions** | | | | | |  |
| Physical security feeling | **Validated (78% quotation > 7)** | |  |  | |  |
| Emotional security feeling | Near consensus, reproposed unaltered | | Emotional security feeling | **Validated 83% quotation > 7** | |  |
| Helplessness feeling | Near consensus, reproposed unaltered | | Feeling of helplessness | Discussed not reproposed | |  |
| Anxiety | **Validated (80% quotation > 7)** | |  |  | |  |
| The feeling of having a future | Discussed, reformulated | | The feeling of being able to project oneself into the future | Discussed not reproposed | |  |
| Relational security feeling | Discussed, not reproposed | |  |  | |  |
| Cognitive security feeling | Discussed, not reproposed | |  |  | |  |
| Environmental security feeling | Discussed, not reproposed | |  |  | |  |
| Psychological security feeling | Near consensus, reproposed unaltered | | Psychological security feeling | Discussed, not validated | |  |
| Sensory well-being | Near consensus, reproposed unaltered | | Sensory well-being | Discussed, not validated | |  |
| Emotional well-being | **Validated (76% quotation > 7)** | |  |  | |  |
| Cognitive well-being | Discussed, not reproposed | |  |  | |  |
| Relational well-being | Near consensus, reproposed unaltered | | Relational well-being | **Validated, 94% quotation > 7** | |  |
| **Scale Item** | | | | | |  |
| I feel in security | **Validated (82% quotation > 7)** | |  |  | | 1 |
| I feel in confidence | **Validated (76% quotation > 7)** | |  |  | | 2 |
| I feel well | Discussed, not reproposed | |  |  | |  |
| I feel comfortable | **Validated (76% quotation > 7)** | |  |  | | 3 |
| I feel serene | Discussed, not reproposed | |  |  | |  |
| I feel calm | Discussed, not reproposed | |  |  | |  |
| I feel human | Discussed, reformulated | | I feel considered as a human (as opposed to a care object) | **Validated, 85% of quotations > 7** | | 8 |
| I feel well informed | **Validated (86% quotation > 7)** | |  |  | | 4 |
| I feel lost | Discussed, not reproposed | |  |  | |  |
| I feel helpless | Discussed, not reproposed | |  |  | |  |
| I feel dependent | Discussed, not reproposed | |  |  | |  |
| I feel worried | Discussed, not reproposed | |  |  | |  |
| I feel anxious | **Validated (76% quotation > 7)** | |  |  | | 6 |
| I feel terrified | Discussed, not reproposed | |  |  | |  |
| I have a positive vision of the future | Discussed, reformulated | | I can project myself into the future | Discussed, not reproposed | |  |
| I can concentrate | Discussed, not reproposed | |  |  | |  |
| I have difficulties understanding what is expected of me | Discussed, not reproposed | |  |  | |  |
| I don't remember what I am told | Discussed, not reproposed | |  |  | |  |
| I'm losing track of my thoughts | Discussed, not reproposed | |  |  | |  |
| I'm having trouble keeping up with conversations | Discussed, not reproposed | |  |  | |  |
| I have difficulties remembering | Discussed, not reproposed | |  |  | |  |
| I am frustrated not being able to communiquate | **Validated (78% quotation > 7)** | |  |  | | 7 |
| I want to be involved in my medical care | Discussed, reformulated | | I want to be informed about the care and medical decisions concerning me | **Validated, 94% of quotation > 7** | | **5** |
|  |  |  | I wish to give my opinion on the care and medical decisions concerning me | **Validated, 75% of quotation > 7** | | **9** |
| **Maximum number of items** | | | | | |  |
| Indicate the maximum number of item the scale may comprise from 3 to 100 (open question) | Minimum: 3  Maximum: 100  1^st^ quartile: 6  Median: 10  3^rd^ quartile: 15 | | **Multiple choice between values below the median maximum** | 1 to 3 questions | 2% |  |
|  |  |  |  | **4 to 5 questions** | **44% Validated** |  |
|  |  |  |  | 6 to 8 questions | 18% |  |
|  |  |  |  | 9 to 10 questions | 31% |  |
| **Response procedure** | | | | | |  |
| Dichotomous Yes/No response | Discussed, proposed altered | | Yes/No response followed by visual numerical scale if Yes. | Validated (78% quotation > 7). | |  |
| Visual numerical scale | Near consensus, discussed proposed altered | |  |  |  |  |
| Color palette | Discussed, not reproposed | |  |  | |  |
| Likert scale “Always/Frequently/Sometimes/Rarely/Never” | Discussed, not reproposed | |  |  | |  |
| Likert scale  “Enormously/A lot/A Little/Absolutely not” | Discussed, not reproposed | |  |  | |  |
| **Administration frequency** | | | | | |  |
| Once a day | Discussed, reformulated | | At least once a day | Not reproposed | |  |
| Twice a day | Discussed, reformulated | | At least twice a day | Validated after discussion (only 70% quotation > 7) | |  |
| Three times a day | Discussed, reformulated | |  |  |  |  |
| Four times a day | **Discussed not reproposed** | |  |  | |  |
| No nighttime administration | **Discussed not reproposed** | |  |  | |  |
| 1^st^ and 2^nd^ Delphi rounds: Except for the population definition which was evaluated in a dichotomous agree/disagree way followed by consensus discussion and the number of item the scale should contain which was evaluated using an open question followed in the second round by multiple choice question between the most frequent values reported in the first round. All other propositions were evaluated using a 1-9 numerical scale. The propositions were validated and not discussed further if at least ¾ of panelist quoted 7 of higher; the propositions were suppressed and not discussed further if more than ½ of panelists quoted below 5. The remaining propositions were discussed, and either reproposed unaltered at the next round if the mean and median quotation was above 7 and more than 70% of panelists quoted above 7 (near favorable consensus) or discussed for potential reformulation for the next round.  In the 3^rd^ Delphi round the panel was asked to classify the 9 item of the scale retained after the 2^nd^ round from the most important (rated 1) to the least important (rated 9). | | | | | |  |

| **Scale validation**   \| The scale used for the last step (Prevalidation group) are presented below:   - **I feel comfortable** o Not at all comfortable o A little comfortable o Comfortable o Very comfortable - **I feel safe** o Not at all safe o A little safe o Safe o Very safe - **I feel well-informed** o Not at all informed o A little informed o Well-informed o Very well-informed - **I feel treated as a person** o Not at all treated as a person o A little treated as a person o Well treated as a person o Very well treated as a person - **I feel anxious** o Not at all anxious o A little anxious o Anxious o Very anxious - **I feel trustful** o Not at all trustful o A little trustful o Trustful o Very trustful \| \| --- \|   **Cohort validation**  The characteristics and the results of the cohort validation are presented in e-Table 5 to e-Table 10.  e-Table 5: Ventilation characteristics for patients at the validation-cohort step   \|  \| First administration (N=84) \| All administrations (N=305) \| \| --- \| --- \| --- \| \| High flow oxygen therapy \| 6 (7%) \| 12 (4%) \| \| Invasive ventilation - Intubated patient \| 66 (79%) \| 136 (45%) \| \| Invasive ventilation - Tracheostomy patient \| 4 (5%) \| 4 (1%) \| \| Non invasive ventilation \| 1 (1%) \| 13 (4%) \| \| Spontaneous ventilation \| 7 (8%) \| 136 (45%) \| \| Not specified \| 0 (0%) \| 4 (1%) \| |
| --- | --- | --- | --- | --- | --- | --- | --- | --- | --- | --- | --- | --- | --- | --- | --- | --- | --- | --- | --- | --- | --- | --- |
| e-Table 6: Estimated completion time considered too long by the caregiver   \|  \| First administration (N=84) \| All administrations (N=305) \| \| --- \| --- \| --- \| \| Yes \| 9 (11%) \| 14 (5%) \| \| No \| 70 (83%) \| 266 (87%) \| \| Not specified \| 5 (6%) \| 25 (8%) \|   e-Table 7: Healthcare professionals’ satisfaction of the scale   \|  \| First administration (N=84) \| All administrations (N=305) \| \| --- \| --- \| --- \| \| Not at all satisfied \| 7 (8%) \| 10 (3%) \| \| A little satisfied \| 20 (24%) \| 49 (16%) \| \| Satisfied \| 42 (50%) \| 177 (58%) \| \| Very satisfied \| 10 (12%) \| 43 (14%) \| \| Not specified \| 5 (6%) \| 26 (9%) \| |
|  |

e-Table 8: Fatigue related to the scale according to the patients

|  | First administration (N=84) | All administrations (N=305) |
| --- | --- | --- |
| Yes | 15 (18%) | 31 (10%) |
| No | 62 (74%) | 240 (79%) |
| Not specified | 7 (8%) | 34 (11%) |

e-Table 9: Estimated completion time considered too long by the patients

|  | First administration (N=84) | All administrations (N=305) |
| --- | --- | --- |
| Yes | 16 (19%) | 31 (10%) |
| No | 61 (73%) | 239 (78%) |
| Not specified | 7 (8%) | 35 (11%) |

e-Table 10: Patients’ satisfaction of the scale

|  | First administration (N=84) | All administrations (N=305) |
| --- | --- | --- |
| Not at all satisfied | 1 (1%) | 4 (1%) |
| A little satisfied | 12 (14%) | 39 (13%) |
| Satisfied | 44 (52%) | 165 (54%) |
| Very satisfied | 14 (17%) | 55 (18%) |
| Not specified | 13 (15%) | 42 (14%) |

*Psychometric characteristics of the initial 6 item scale*

Previously, item 5 was reversed because it is the only item phrased negatively (anxiety) compared to the others (comfort, safety, information, being treated as a person, and trust).

The table below shows the values associated with each response option.

|  | Not at all | A little | well | very |
| --- | --- | --- | --- | --- |
| comfortable | 0 | 1 | 2 | 3 |
| safe | 0 | 1 | 2 | 3 |
| informed | 0 | 1 | 2 | 3 |
| considered | 0 | 1 | 2 | 3 |
| anxious | 3 | 2 | 1 | 0 |
| trustful | 0 | 1 | 2 | 3 |

Values were distributed from 0 to 18 points, with a mean of 12.2 (± 3.1) and a median of 12 (minimum 0, maximum 18). The distribution histogram is presented in eFigure 2. The distribution was even with no important ceiling effect, nor floor effect or asymmetry. The overall reliability of the scale was correct (alpha=78%) but removing item 5 (anxiety assessment) improved it (alpha=85%). Several other metrics pointed towards an issue with this item. Overall data consistency was good (H=0.42) however anxiety assessment had a low consistency with the other items (H_item5_=0.08). Overall it seemed not to fit well in the scale: it was the only item formulated negatively (anxiety), the patients gave on average the most pejorative values (1.38 on average after inversion against 1.89 to 2.42 on average for the others items), and it was the item with the most dispersed values (standard deviation at 0.88 against 0.69 to 0.81 for the other items). Furthermore, the item biplot (e-Figure 3) shows that item 5 seems to measure a dimension that is significantly different from the other items. The confirmatory factor analysis was moderately satisfactory. The factor loading of item 5 was low (0.08<0.4) showing that this item is problematic and does not measure a one-dimensional concept with the others. The CFI and TLI adequacy coefficients were satisfactory (>0.95) but the RMSEA coefficient was too high (0.09>0.08).

eFigure 2: Distribution of the 6-item score across the 305 assessments (284 complete).


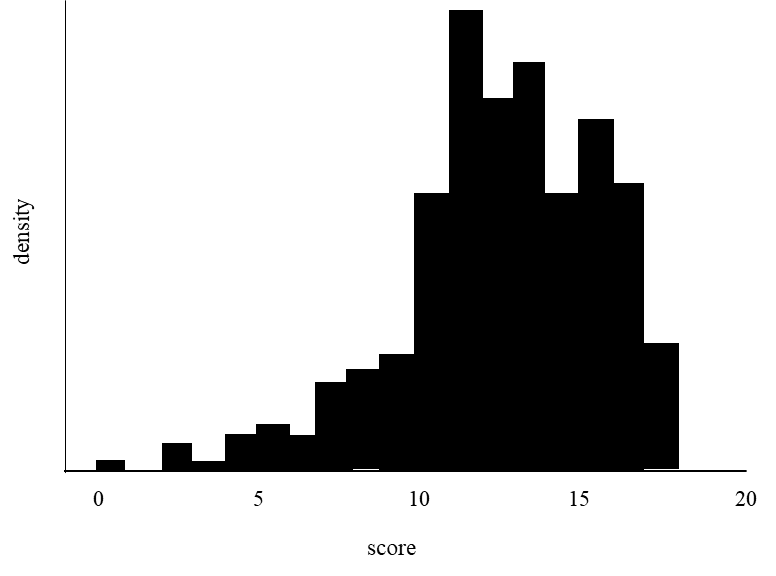


eFigure 3: The items Biplot of the scale with 6 items


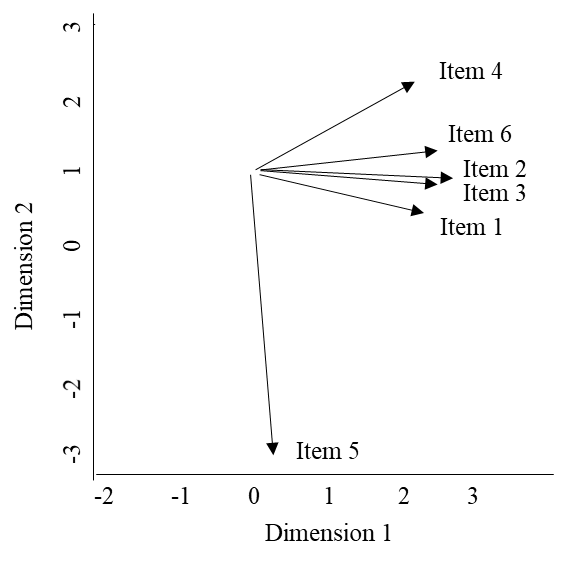


Legend:

Item 1 comfortable/Item 2 safe/Item 3 informed/Item 4 considered/Item 5 anxious/Item 6 trustful

*Psychrometric characteristics of the 5 item scale:*

The distribution histogram is presented in eFigure 4. The reliability was correct (alpha=85%). Data consistency was good (H=0.61). All the items showed a good coherence with the others. The item biplot showed a common general direction measured by the 5 items (e-Figure 5). The confirmatory factor analysis was satisfactory. All the loadings’ factors were homogeneous (between 0.63 and 0.78) showing the interest of each of the 5 items. The CFI and TLI adequacy coefficients were satisfactory (>0.95) but the RMSEA coefficient was high (0.09>0.08).

eFigure 4: Distribution of the 5-item score across the 305 assessments (284 complete).


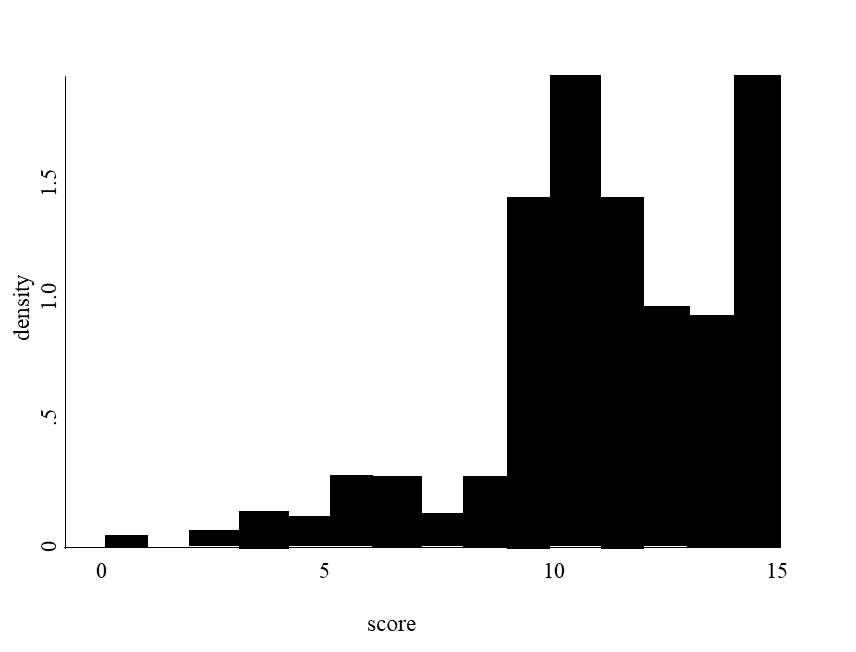


e-Figure 5: The items Biplot of the scale with 5 items


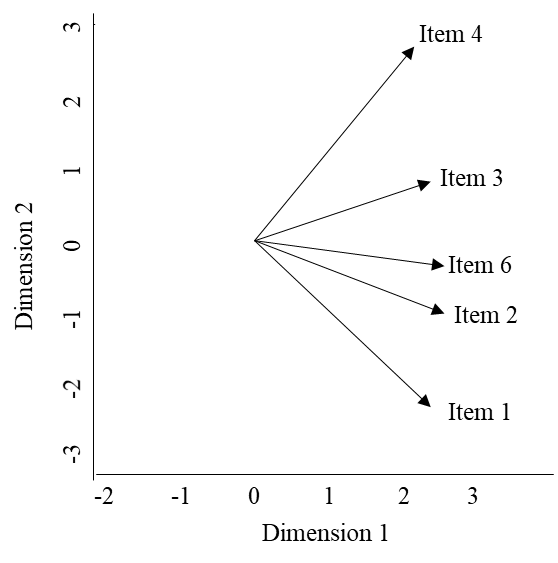


Legend: Item 1 comfortable/Item 2 safe/Item 3 informed/Item 4 considered/Item 6 trustful
